# Research priorities for faecal incontinence in adults: A James Lind Alliance priority setting partnership

**DOI:** 10.1111/codi.70154

**Published:** 2025-07-10

**Authors:** Alexander O'Connor, Sam Alexandra Rose, Emma V. Carrington, Anna Clements, Julie A. Cornish, Marcus J. Drake, Louise J. Dunford, Jennie Grainger, Douglas Hallett, Kate Lough, Tatenda Marunda, Aziza Sesay, Dipesh H. Vasant, Tara Willson, Thomas Dudding

**Affiliations:** ^1^ Department of Colorectal Surgery Manchester University NHS Foundation Trust Manchester UK; ^2^ Faculty of Biology, Medicine and Health University of Manchester Manchester UK; ^3^ Manchester Academic Health Sciences Centre Manchester UK; ^4^ The Pelvic Floor Society London UK; ^5^ Bowel Research UK London UK; ^6^ Department of Surgery and Cancer Imperial College London London UK; ^7^ Imperial College Healthcare NHS Trust London UK; ^8^ MASIC Charity UK; ^9^ Department of General Surgery Cardiff and Vale University Health Board Cardiff UK; ^10^ Warwick Medical School University of Warwick Coventry UK; ^11^ James Lind Alliance University of Southampton Southampton UK; ^12^ Department of Colorectal Surgery Countess of Chester Hospital NHS Foundation Trust Chester UK; ^13^ Pelvic Obstetric and Gynaecological Physiotherapy (POGP) Organisation UK; ^14^ St Mark's National Bowel Hospital London UK; ^15^ Palfrey Health Centre, National Health Service Walsall UK; ^16^ Neurogastroenterology Unit, Wythenshawe Hospital Manchester University NHS Foundation Trust Manchester UK; ^17^ University Hospital Southampton NHS Foundation Trust Southampton UK

**Keywords:** faecal incontinence, James Lind Alliance, research, research priorities, research prioritisation

## Abstract

**Aim:**

Faecal incontinence (FI) is common, yet clinical guidelines rely on low‐quality evidence or expert opinion. A high proportion of research is focused on areas that may not be considered a priority by patients or clinicians. This project aimed to identify the top 10 research priorities for FI in adults in equal collaboration with patients, carers and healthcare professionals in a James Lind Alliance priority setting partnership (PSP).

**Methods:**

This PSP followed established methodology supported by a multidisciplinary steering group including those with lived experience of FI. Evidence uncertainties were gathered through a survey with free‐text responses, summarised in indicative summary questions and prioritised in a second survey. An independently facilitated priority setting workshop used a nominal group technique to reach consensus on the order of research priorities, with a focus on the top 10. At all stages the views of healthcare professionals and individuals with a lived experience of FI were considered equally.

**Results:**

After the initial survey, 512 respondents submitted 991 evidence uncertainties. These produced 54 indicative summary questions. In the second survey, 373 respondents generated a shortlist of 26 questions. Finally, the top 10 research priorities were determined by consensus at a face‐to‐face workshop and include unanswered questions concerning prevention, investigation, education, self‐management and treatment of FI.

**Conclusion:**

This PSP has identified a comprehensive list of top research priorities, including items of importance to both healthcare professionals and individuals with a lived experience of FI. Researchers and funders should use these priorities to inform future work.

**What does this paper add to the literature?**

Faecal incontinence is common, yet research is lacking without overall strategic direction. This project brought together patients, carers and healthcare professionals in equal partnership to define the top 10 research priorities for faecal incontinence in adults. This provides a strong basis for future research underpinned by robust patient and public involvement.

## INTRODUCTION

Faecal incontinence (FI) affects approximately 8% of adults and, whilst it affects all ages and both genders, the prevalence is greatest amongst women and older people [1]. It is commonly defined as the involuntary loss of liquid or solid stool which is a social or hygienic problem, and it carries a significant impact on an individual's quality of life [2, 3]. However, people with FI also frequently report faecal urgency and flatus incontinence which add to their debilitating symptoms [2]. Despite the impact on quality of life, patients may not volunteer their symptoms to a healthcare professional owing to embarrassment and the associated ‘taboo’ [4, 5]. FI has therefore been described as a ‘silent affliction’, with sufferers experiencing social isolation and anxiety as they live their lives close to a toilet [4]. This in turn leads to negative effects on mental health, employment, relationships and daily activities.

The aetiology of FI is multi‐factorial and often attributed to anatomical disturbances of the anorectum and pelvic floor, anorectal dysfunction, uncontrolled diarrhoea and neurological disorders [3, 6]. Whilst most patients can be managed in the community with conservative interventions, those with persistent or severe symptoms may require further investigation and management in secondary or tertiary care [7, 8, 9]. National clinical guidelines have been published for FI management; however, their recommendations frequently rely on low‐quality evidence or expert opinion, with uncertainties around the correct treatment approach for the individual patient [7, 10]. In addition, there are geographical disparities with variable access to specialist services across the UK, further compounding the issues and lengthening the treatment pathway for those with FI [11, 12].

Research into FI is limited compared to other diseases [13, 14] and is primarily focused on its investigation and management in a hospital setting. In recent years, researchers and national funding bodies have advocated for patient involvement in setting the research agenda. The James Lind Alliance (JLA) is a not‐for‐profit initiative hosted by the National Institute for Health Research with the aim of bringing together patients, carers and clinicians in a priority setting partnership (PSP) [15]. Each JLA PSP seeks to identify and prioritise important unanswered questions about a particular health condition and agree a list of top 10 research priorities. The JLA methodology has been refined since 2004 and used for a wide range of conditions. It emphasises an equal partnership between patients, carers and clinicians in reaching agreement on the research priorities [16]. Results from PSPs are then often used by researchers and their funders to increase research activity and funding allocation [17].

Despite the prevalence of FI, and the impact on an individual's life, the priorities for future FI research are unknown. Our aim, therefore, was to use the JLA methodology to bring together, for the first time, patients, carers and healthcare professionals to define the top 10 research priorities for FI.

## METHOD

### James Lind Alliance methodology

This project followed the *JLA Guidebook* (version 10.0), first published in March 2021 [16], and was reported following the Reporting Guideline for Priority Setting of Health Research [18].

### Setting up the priority setting partnership

A steering group (SG) was formed in September 2023 consisting of 16 members. Initially, four members of the Pelvic Floor Society executive team and one pelvic floor research fellow established the PSP after liaising with the JLA coordinating office. The PSP was then allocated a JLA advisor, who provided guidance and support throughout the process and ensured the PSP was conducted in accordance with JLA methodology (LD). The remaining SG members were recruited with consideration to the multidisciplinary approach taken to FI management. Leaders of key professional organisations and bowel charities were contacted to participate in the PSP or identify suitable experienced FI healthcare professionals and patients who would be willing to participate. With the exception of the JLA advisor, no SG member received reimbursement for their time. In total the SG included four patients (three women, one man) and 11 healthcare professionals (four colorectal surgeons, urologist, general practitioner, gastroenterologist, specialist physiotherapist, advanced biofeedback practitioner, specialist nurse and research fellow). In addition, an experienced information specialist was recruited to support the PSP with data synthesis and analysis of survey results (TG). All members had links to wider networks, charities and professional organisations to promote the PSP.

### Scope of the priority setting partnership

The PSP considered the definition of FI to include the uncontrolled loss of liquid or solid stool in addition to the uncontrolled loss of flatus, faecal urgency, the fear or anxiety of incontinence. The PSP also included within the definition of FI situations where continence is only maintained through modifications to a patient's lifestyle (e.g., remaining at home or near toilet facilities).

The agreed scope of this PSP was broad and considered questions about FI in adults (>18 years) related to epidemiology, aetiology, investigation and management. In addition, questions were considered related to the effect of FI on patients' and carers' lives and the barriers experienced by them in accessing support and treatment. Questions about the management of FI under paediatric care and FI associated with inflammatory bowel disease or chronic diarrhoea were considered out of scope and were excluded.

### Gathering evidence uncertainties

In March 2024 an online survey was launched in the UK entitled ‘Gathering evidence uncertainties’ at the House of Lords in the UK Parliament. The survey was hosted on the Qualtrics™ survey platform (Qualtrics, UT, USA) and shared using QR codes and short URL links.

The aim was to collect unanswered questions about FI from patients, carers and health and social care professionals using a free‐text response to the following open question.

**Based on your experiences, what problems or needs should future research address to improve the experience of people living with faecal incontinence and/or those close to them?**

These could include, but are not limited to, who suffers with faecal incontinence and why, the investigations, tests or treatments for faecal incontinence, the physical, psychological or social support for patients and/or those close to them, or the barriers experienced by patients and/or those close to them in accessing support for faecal incontinence.

They may be related to something you, your loved ones or patients are currently experiencing, something you wish you had known earlier, something you are unsure about in the future or something you wish was generally known about.

In addition to providing an answer to this question, respondents were asked to indicate whether they were a person living with FI, a carer, a social care professional or a healthcare professional. Demographic data were collected solely for the purpose of checking that the survey had been completed by a diverse range of participants, and these questions were optional.

In designing this survey, the SG considered other active and recently completed JLA surveys. In addition, our survey was piloted amongst the SG of patients and healthcare professionals. The survey remained open for 6 weeks and was publicised by several leading bowel charities (e.g., Bowel Research UK, MASIC, Bladder and Bowel UK, Guts UK) and professional organisations (e.g., Pelvic Floor Society, Association of Continence Professionals, Pelvic Obstetric and Gynaecological Physiotherapy), with extensive patient and healthcare professional memberships. Social media platforms were used to further promote the survey in patient and healthcare professional groups, whilst SG members distributed the survey amongst their own networks. The SG reviewed survey responses and targeted specific under‐represented groups to encourage involvement where necessary.

### Summarising the responses

A professional JLA information specialist (TG) with experience of the qualitative, thematic analysis methodology advocated by the JLA was recruited. This information specialist analysed each written response from the ‘gathering evidence uncertainties’ survey. Multiple questions or uncertainties from a single respondent were now separated into individual submissions. These individual questions or uncertainties were then categorised into key themes by the information specialist. Next, working with a data subgroup, consisting of four healthcare professionals and two patients from the main SG, responses in each theme were reviewed. Using their clinical and lived experience of FI, they generated indicative summary questions covering each identified theme. As with all other JLA PSPs, each summary question was considered to reflect the group of responses within a theme and were not necessarily written as precise ‘research questions’ but rather captured the ideas and concerns of the respondents. Responses considered out of scope were removed and any disagreements were referred for discussion within the main SG.

Once the indicative summary questions were agreed, two SG members (AOC and TD) ensured they had not already been answered by previous research. National guidelines were reviewed and the Cochrane Database of Systematic Reviews, PubMed, MEDLINE, Embase and Google Scholar were searched for relevant systematic reviews published since 2020. Questions which were not wholly answered by the available evidence were used in the subsequent (interim priority setting) survey.

### Interim priority setting

A second interim priority setting ‘shortlisting’ survey was launched in July 2024 and distributed using the same networks. Respondents from the first survey who provided their contact details and had indicated a willingness to continue their involvement were also invited to complete this second survey. The aim of the second survey was to select a shorter list of indicative summary questions to be discussed at the final priority setting workshop.

Respondents were presented with a list of all indicative summary questions in a random order and asked to select ‘up to 10 questions that are important to you’ from this list. Respondents could select any number of questions between 1 and 10. As with the first survey, respondents were required to indicate whether they were a person living with FI, a carer, a social care professional or a healthcare professional. Providing further demographic data was optional and used to check that data were being collected from a heterogeneous group of participants.

Responses were split into two groups: healthcare and social care professionals and individuals with a lived experience of FI (people living with FI, carers and those who preferred not to declare). The summary questions for each group were ranked according to the number of respondents who selected each question. To ensure equal influence of both groups according to the JLA principles, the rank of each question was combined from both groups to create a weighted rank (healthcare and social care professionals rank + individuals with a lived experience of FI rank = weighted rank). Using this approach, the views of both groups were weighted equally regardless of the total number of responses received from each. The highest ranked questions, including the top 10 ranked questions from each group, were then taken forward for discussion at a final workshop after ratification by the SG.

### Final priority setting workshop

The final face‐to‐face workshop took place on 4 October 2024 at the Royal College of Surgeons of England, London. The workshop was chaired by three independent JLA advisors. Twenty‐eight participants attended (13 patients and 15 healthcare professionals) with travel costs reimbursed. The composition of the workshop participants ensured there was equal representation from a diverse group of patients, including male and female individuals with different FI aetiologies and healthcare professional groups across different geographical locations in the UK.

All participants were given a list of the questions from the interim priority setting survey prior to the meeting and asked to rank them individually before attending the workshop. At the workshop participants were split into three groups, with each group containing patients with lived experience and healthcare professionals, facilitated by a JLA adviser. During the first session participants were asked to share their individual top three and bottom three questions, and their reasons for choosing these. In the second session participants were asked to rank all questions in order as a group, coming to an agreement by consensus, with the JLA advisor ensuring all voices in the group were heard using a nominal group technique [19].

The results from the three groups were then combined to create an aggregate ranking. In the third session the groups were reallocated, so individuals had the opportunity to hear different perspectives, and the process of ranking by consensus was repeated. Finally, the overall aggregate ranking was presented to the whole group who reached a consensus on the final order with a particular focus on the top 10 research priorities.

Following the workshop, the final results will be communicated to the bowel charities and other organisations involved to distribute amongst their networks of patients and healthcare professionals. Researchers and funders will then be made aware of the findings and encouraged to integrate the results into their decision making processes around funding allocation.

### Ethics statement

This was a patient and public involvement in research project which did not collect identifiable information. The individuals who took part in this project were not research participants. In line with the UK Health Research Authority decision tool there is not a requirement for ethics approval, consistent with other JLA PSP projects.

## RESULTS

**FIGURE 1:**
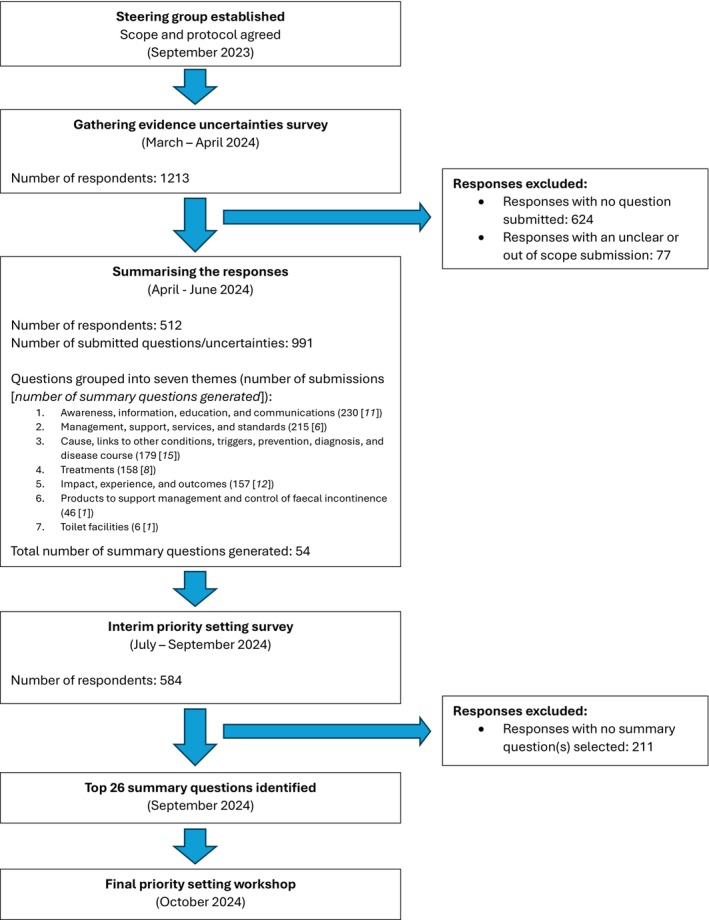
A summary of the stages of the priority setting partnership.

A summary of the process and timeline of the PSP project is presented in Figure 1.

### Gathering evidence uncertainties

After distribution of the survey a total of 1213 individuals responded by accessing the online survey platform. Of these, 512 provided at least one written response that was within the scope of the PSP.

The demographic characteristics of the 512 respondents are summarised in Table 1. More patients with a lived experience (60%) responded than healthcare professionals (33%) and there was a predominance of white women aged between 41 and 60. From these respondents, 991 individual questions or evidence uncertainties about FI were submitted and reviewed by the data subgroup.

**TABLE 1:** Demographic characteristics of the survey respondents.

Demographic *n* (%)	Gathering evidence uncertainties survey (*n* = 512)	Interim priority setting survey (*n* = 373)
Respondent category		
Healthcare professional	169 (33)	139 (37)
Patient with a lived experience of faecal incontinence	308 (60)	217 (58)
Family, friend or informal caregiver	26 (5)	11 (3)
Other/not stated	9 (2)	6 (2)
Age		
<20	1 (<1)	1 (<1)
21–40	79 (15)	56 (15)
41–60	217 (42)	162 (43)
61–80	181 (35)	127 (34)
>80	19 (4)	16 (4)
Not stated	15 (3)	11 (3)
Gender		
Male	87 (17)	57 (15)
Female	415 (81)	307 (82)
Non‐binary	4 (1)	0 (0)
Not stated	6 (1)	9 (2)
Heritage		
White	450 (88)	325 (87)
Black	6 (1)	4 (1)
Asian	30 (6)	21 (6)
Mixed	11 (2)	9 (2)
Other/not stated	15 (3)	14 (4)

### Summarising the responses

Following removal of the responses considered out of the scope of the PSP (*n* = 77), the remaining 991 individual submissions were grouped into seven key themes (Figure 1). These submissions were reviewed by the data subgroup and used to generate 54 indicative ‘summary questions’ across each theme (Table S1). None of these questions had been sufficiently answered by previous research to the threshold set out by the JLA to be excluded (systematic review or guidelines) and they were therefore all taken forward to the interim priority setting survey.

### Interim priority setting

A total of 584 individuals responded to the interim priority setting survey and, of these, 373 respondents selected at least one of the 54 summary questions important to them. The demographic characteristics of the survey respondents were similar to those from the first survey (Table 2). The majority (*n* = 291, 78%) selected 10 questions from the available list of 54, with respondents submitting an average of nine questions.

**TABLE 2:** The top 26 summary questions presented to the final priority setting workshop.

Summary question	Lived experience rank	HCP rank	Weighted rank^a^
How effective are pelvic floor exercises to prevent, reduce or control FI?	6	2	8
What are the most effective and acceptable treatment options for a given individual living with FI?	3	5	8
What is the nature of the connection between the brain–gut axis and FI? (the brain–gut axis is a two‐way communication network that links the bowel and brain)	10	3	13
What part does the community of microbes in the gut (the gut microbiome) play in FI?	14	4	18
Why does FI seem to be triggered by stress and anxiety, and how can this be managed?	12	7	19
What are the most effective methods to prevent or control faecal urgency in people living with FI?	1	19	20
What is the role of neurostimulation/neuromodulation treatments in FI and faecal urgency? (e.g., sacral nerve stimulation (SNS) or tibial nerve stimulation (PTNS/TTNS))	19	1	20
What new surgical techniques for FI could be developed and which existing surgeries could be improved to be less invasive?	9	13	22
What are the most effective and acceptable tests to accurately understand the cause of FI and inform relevant treatments?	17	12	29
What is the role of medications in treating FI, and how can the range of medications be improved?	7	23	30
How can the risks of FI associated with surgery, pelvic health, cancer treatment and childbirth be effectively communicated to patients, so there is understanding and informed agreement?	21	9	30
Which foods, supplements or medications are triggers for FI, and are these specific to each person?	5	28	33
What new and novel approaches to managing FI could be developed? (e.g., stem cells or bioelectronics)	25	11	36
How can people living with FI be empowered to seek professional help and receive guidance to manage their condition in a safe and effective way? (e.g., exercises, medications, dietary advice and hydration)	16	20	36
How are GPs and other primary HCPs best trained and supported to provide best care for people living with FI?	15	25	40
How can the psychological impact of FI be better understood?	13	29	42
What is the cause of flatus incontinence (uncontrolled passage of wind) and how can it best be managed?	11	32	43
How can the common causes of FI be better understood, predicted and prevented?	29	14	43
How can worsening or deterioration of FI be predicted and prevented? (e.g., due to ageing or childbirth)	22	22	44
How effective are current FI products (e.g., pads and plugs), and are they sufficiently adaptable for an individual's needs?	20	27	47
What is the role of lifestyle factors in causing FI? (e.g., obesity, mental health and anal sex)	39	8	47
What is the impact of needing to access public toileting facilities frequently and/or urgently on people living with FI?	2	48	50
How can provision of and access to FI services and products across the UK be standardised?	40	10	50
In post‐menopausal women, what is the role of topical HRT in preventing FI? (e.g., creams, ointments, pessaries and patches)	46	6	52
What is the impact of FI on an individual's social and day‐to‐day life?	4	50	54
What would be the impact of recognising FI as a disability on the wellbeing of people living with FI?	8	53	61

*Note*: 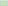
Green background, question featured in both lived experience and HCP top 10; 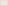
orange background, question featured in HCP top 10; 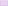
purple background, question featured in lived experience top 10.

Abbreviations: FI, faecal incontinence; GP, general practitioner; HCP, healthcare professional; HRT, hormone replacement therapy; PTNS, percutaneous tibial nerve stimulation; SNS, sacral nerve stimulation; TTNS, transcutaenous tibial nerve stimulation.

^a^ Weighted rank calculated as lived experience rank + HCP rank.

There were notable differences in the priorities identified by respondents with a lived experience of FI, and healthcare professionals. Only three questions were common to the top 10 rankings from the two groups (Table 2).

Questions were ordered according to the weighted rank, and it was suggested by the JLA advisor that a maximum of 26 questions should be taken forward to the final priority setting workshop. Therefore, those selected included the top 10 questions from each group, including the three questions in both groups, and the next nine highest ranked questions (Table 2).

### Final priority setting workshop

The final workshop was attended by 28 individuals. The workshop was observed by members of the SG and facilitated by three JLA advisors.

The final top 10 research priorities are presented in Table 3 (the ranks of all 26 questions are presented in Table S2). The final top 10 research priorities agreed at the workshop differed from the results of the interim priority setting survey, with just five featuring in either the lived experience or healthcare professional top 10. Further, two questions that featured in the top 10 of both groups from the interim survey did not feature in the overall results (‘How effective are pelvic floor exercises to prevent, reduce or control FI?’ and ‘What is the nature of the connection between the brain–gut axis and FI?’).

**TABLE 3:** The top 10 research priorities for faecal incontinence in adults.

Rank	Question
**1**	**How are GPs and other primary healthcare professionals best trained and supported to provide best care for people living with FI?**
**2**	**How can the common causes of FI be better understood, predicted and prevented?**
**3**	**How can the risks of FI associated with surgery, pelvic health, cancer treatment and childbirth be effectively communicated to patients, so there is understanding and informed agreement?**
**4**	**How can people living with FI be empowered to seek professional help and receive guidance to manage their condition in a safe and effective way? (e.g., exercises, medication, dietary advice and hydration)**
**5**	**What are the most effective and acceptable treatment options for a given individual living with FI?**
**6**	**How can worsening or deterioration of FI be predicted and prevented? (e.g., due to ageing or childbirth)**
**7**	**What are the most effective methods to prevent or control faecal urgency in people living with FI?**
**8**	**What is the role of medications in treating FI, and how can the range of medications be improved?**
**9**	**How can the psychological impact of FI be better understood?**
**10**	**What part does the community of microbes in the gut (the gut microbiome) play in FI?**

Abbreviations: Fi, faecal incontinence; GP, general practitioner.

## DISCUSSION

This PSP followed the rigorous JLA methodology that has been used to define research priorities for over 100 other health conditions and diseases [20] and was guided by a SG that included patients with a lived experience of FI and healthcare professionals. By considering the views of over 900 individuals it has identified a top 10 list of priorities covering a broad range of research uncertainties including the prevention, investigation, education, self‐management and treatment of FI across primary, secondary and tertiary care. At each stage of the PSP process, the contributions of patients and healthcare professionals were equally weighted to represent their different priorities. This has led to consensus on a diverse list of research priorities that considers the views of all groups. These research priorities should now be communicated to research funders and stakeholders involved in this process and be integrated into the decision making for funding allocation.

FI is typically defined as the involuntary loss of liquid or solid stool [3]. However, patients with faecal urgency have symptoms that are equally debilitating, often provoking anxiety and limiting daily activities due to the fear of experiencing incontinence [2]. The definition of FI used in this PSP was therefore deliberately broad and included flatus incontinence, faecal urgency and situations where continence is only preserved through a modification to a patient's lifestyle. It is notable, therefore, that a top 10 research priority considers the most effective method to prevent and control faecal urgency. By contrast, the question concerning flatus incontinence was the lowest ranked at the priority setting workshop despite this symptom featuring in the FI severity measures commonly used in clinical practice [21, 22]. This highlights the importance and validity of patient participation in directing research in FI.

The final priority setting workshop encouraged thoughtful and passionate exchanges between different groups when considering the rank of each indicative summary question. Discussions during the workshop were notable in their efforts to include broad questions such as ‘How can the common causes of FI be better understood, predicted and prevented?’ and ‘What are the most effective and acceptable treatment options for a given individual living with FI?’ above specific questions, such as those concerning novel topics (e.g., stem cells), or questions with an anticipated limited impact on clinical practice. Based on the established nominal group technique used in the JLA methodology all views are considered equally to build a consensus agreement on the final top 10 priorities [19]. For example, the top priority identified by healthcare professionals following the interim priority setting survey, considering the role of neuromodulation therapies, did not feature in the final top 10 after the workshop. This was influenced by the perceived breadth of research already being undertaken in this area. Similar differences in question ranks between the interim survey and the final workshop have been observed in other PSPs [23, 24]. This may be explained by the use of facilitated group discussions which are considered an effective way to reach consensus, since potential conscious or unconscious biases, which influence individual decision making, are made explicit and overcome [25]. As a further example of this effect, the top question identified at the workshop ‘How are general practitioners and other primary healthcare professionals best trained and supported to provide best care for people living with FI?’ was previously ranked 15th and 25th by patients and healthcare professionals respectively following the interim priority setting survey but was ranked first during the workshop after the attendees acknowledged the importance of general practitioners as gatekeepers to further support and management of FI.

To address all the research priorities identified in this PSP will require various methodological approaches including qualitative research, basic science studies and interventional trials, which may be challenging given the breadth of the priorities. However, it is the broad nature of these research priorities, rather than specific research questions, that presents opportunities for researchers and funders to develop proposals that address one or more of these priorities. The outcome of these studies could then produce results relevant to all healthcare settings.

Whilst this PSP has for the first time considered the views of both patients and healthcare professionals in directing the research agenda in FI, we acknowledge there are limitations. These may include the limited views obtained from specific groups including men, ethnically diverse communities and carers, despite the efforts of the SG. In addition, the surveys were only available online, which may have been a barrier to participation in low socioeconomic communities. Finally, the priority setting workshop was held face‐to‐face in London which may have disincentivised individuals with socially limiting FI to attend, although it was felt moving to an online or hybrid platform would have negatively impacted the dynamic discussions of the workshop.

## CONCLUSION

This PSP has provided an authoritative list of research priorities for FI to the academic community and its funders with a focus on topics important to both patients with a lived experience of FI and healthcare professionals involved in their care. The top 10 priorities provide opportunities to develop a wide range of proposals across qualitative research, basic science studies and interventional trials underpinned by patient and public involvement. The challenge remains to encourage researchers to address these priorities, and to influence funding bodies, but in doing so there is the potential to significantly improve the lives of those individuals suffering with FI.

## AUTHOR CONTRIBUTIONS

**Alexander O'Connor:** Writing – original draft; methodology; validation; data curation; project administration; formal analysis; investigation. **Sam Alexandra Rose:** Validation; writing – review and editing; formal analysis; data curation; investigation. **Emma V. Carrington:** Writing – review and editing; visualization; validation; methodology; formal analysis; supervision; investigation. **Anna Clements:** Data curation; writing – review and editing; formal analysis; investigation. **Julie A. Cornish:** Data curation; supervision; formal analysis; project administration; validation; visualization; methodology; writing – review and editing; resources; investigation. **Marcus Drake:** Writing – review and editing; methodology; validation; investigation. **Louise J. Dunford:** Supervision; conceptualization; writing – review and editing; methodology; project administration; validation. **Jennie Grainger:** Formal analysis; data curation; writing – review and editing; investigation. **Douglas Hallett:** Methodology; writing – review and editing; formal analysis; investigation; data curation. **Kate Lough:** Resources; data curation; formal analysis; methodology; writing – review and editing; validation; investigation. **Tatenda Marunda:** Data curation; formal analysis; writing – review and editing; validation; visualization; methodology; resources; investigation. **Aziza Sesay:** Resources; writing – review and editing; visualization; methodology; investigation. **Dipesh H. Vasant:** Methodology; validation; writing – review and editing; investigation. **Tara Willson:** Investigation; methodology; writing – review and editing; data curation; formal analysis. **Thomas Dudding:** Conceptualization; funding acquisition; supervision; resources; project administration; methodology; validation; writing – original draft.

## FUNDING INFORMATION

This study was funded by an unconditional research grant from the Pelvic Floor Society. AOC received funding from The Royal College of Surgeons of England (Award/Grant numbers are not applicable).

## CONFLICT OF INTEREST STATEMENT

Sam Alexandra Rose, Emma V. Carrington, Anna Clements, Louise J. Dunford, Jennie Grainger, Douglas Hallett, Kate Lough, Tatenda Marunda, Aziza Sesay, Dipesh H. Vasant, Tara Willson wish to declare no conflicts of interest. Alexander O'Connor received grant funding from the Royal College of Surgeons of England during this work. Julie A. Cornish has received grants/research support from BD and Medtronic, honoraria from Qufora, and has participated as a speaker at an event sponsored by Coloplast. Marcus Drake is a trustee of the International Continence Society. Thomas Dudding has received grants/research support and honoraria from Medtronic and Axonics.

## PATIENT CONSENT STATEMENT

Participants were not required to provide informed consent to participate in this patient and public involvement in research project.

## Supporting information


Data S1.

## Data Availability

The data presented in this manuscript are available from the corresponding author on reasonable request.

## References

[1] Mack I , Hahn H , Godel C , Enck P , Bharucha AE . Global prevalence of fecal incontinence in community‐dwelling adults: a systematic review and meta‐analysis. Clin Gastroenterol Hepatol. 2024;22(4):712–731.e8. 37734583 10.1016/j.cgh.2023.09.004 PMC10948379

[2] Bharucha AE , Knowles CH , Mack I , Malcolm A , Oblizajek N , Rao S , et al. Faecal incontinence in adults. Nat Rev Dis Primers. 2022;8(1):53. 35948559 10.1038/s41572-022-00381-7

[3] Heitmann PT , Vollebregt PF , Knowles CH , Lunniss PJ , Dinning PG , Scott SM . Understanding the physiology of human defaecation and disorders of continence and evacuation. Nat Rev Gastroenterol Hepatol. 2021;18(11):751–769. 34373626 10.1038/s41575-021-00487-5

[4] Johanson JF , Lafferty J . Epidemiology of fecal incontinence: the silent affliction. Am J Gastroenterol. 1996;91(1):33–36. 8561140

[5] Brown HW , Guan W , Schmuhl NB , Smith PD , Whitehead WE , Rogers RG . If we don't ask, they won't tell: screening for urinary and fecal incontinence by primary care providers. J Am Board Fam Med. 2018;31(5):774–782. 30201674 10.3122/jabfm.2018.05.180045 PMC6170156

[6] Rasijeff AMP , Garcia‐Zermeno K , Di Tanna GL , Remes‐Troche J , Knowles CH , Scott MS . Systematic review and meta‐analysis of anal motor and rectal sensory dysfunction in male and female patients undergoing anorectal manometry for symptoms of faecal incontinence. Color Dis. 2022;24(5):562–576. 10.1111/codi.16047 PMC9303800 35023242

[7] Assmann SL , Keszthelyi D , Kleijnen J , Anastasiou F , Bradshaw E , Brannigan AE , et al. Guideline for the diagnosis and treatment of faecal incontinence—a UEG/ESCP/ESNM/ESPCG collaboration. United European Gastroenterol J. 2022;10(3):251–286. 10.1002/ueg2.12213 PMC9004250 35303758

[8] Rao SS , Benninga MA , Bharucha AE , Chiarioni G , Di Lorenzo C , Whitehead WE . ANMS‐ESNM position paper and consensus guidelines on biofeedback therapy for anorectal disorders. Neurogastroenterol Motil. 2015;27(5):594–609. 25828100 10.1111/nmo.12520 PMC4409469

[9] Paquette IM , Varma MG , Kaiser AM , Steele SR , Rafferty JF . The American Society of Colon and Rectal Surgeons' clinical practice guideline for the treatment of fecal incontinence. Dis Colon Rectum. 2015;58(7):623–636. 26200676 10.1097/DCR.0000000000000397

[10] NICE . Faecal incontinence in adults: management CG49: National Institute for Heath and Care Excellence. 2007. Available from: https://www.nice.org.uk/guidance/cg49 39480986

[11] TPFS . Seizing the opportunity to improve patient care: pelvic floor services in 2021 and beyond. The Pelvic Floor Society. 2021 [updated 2021]. Available from: https://www.pelvicfloorreport.com/ 10.12968/hmed.2021.0387 34601937

[12] O'Connor A , Mullins D , Sharma A , Faulkner G , Telford K . Sacral neuromodulation: time to seize the opportunity to collaborate on a ‘de‐prioritised’ service? Tech Coloproctol. 2023;27(6):517–518. 37004658 10.1007/s10151-023-02785-3 PMC10066949

[13] BRUK . The “Hard to Fund” Fund: Bowel Research UK. Available from: https://www.bowelresearchuk.org/our‐research/the‐hard‐to‐fund‐fund/#:~:text=The%20new%20“Hard%20to%20Fund,Rarer%20conditions%20or%20diseases

[14] UK Clinical Research Collaboration . UK Health Research Analysis 2022. 2023. Available from: https://hrcsonline.net/reports/analysis‐reports/uk‐health‐research‐analysis‐2022/

[15] Nygaard A , Halvorsrud L , Linnerud S , Grov EK , Bergland A . The James Lind Alliance process approach: scoping review. BMJ Open. 2019;9(8):e027473. 10.1136/bmjopen-2018-027473 PMC6720333 31473612

[16] JLA . JLA Guidebook 2021. Available from: https://www.jla.nihr.ac.uk/jla‐guidebook

[17] Staley K , Crowe S , Crocker JC , Madden M , Greenhalgh T . What happens after James Lind Alliance priority setting partnerships? A qualitative study of contexts, processes and impacts. Res Involv Engagem. 2020;6:41. 32670611 10.1186/s40900-020-00210-9 PMC7353437

[18] Tong A , Synnot A , Crowe S , Hill S , Matus A , Scholes‐Robertson N , et al. Reporting Guideline for Priority Setting of Health Research (REPRISE). BMC Med Res Methodol. 2019;19(1):243. 31883517 10.1186/s12874-019-0889-3 PMC6935471

[19] Gallagher M , Hares T , Spencer J , Bradshaw C , Webb I . The nominal group technique: a research tool for general practice? Fam Pract. 1993;10(1):76–81. 8477899 10.1093/fampra/10.1.76

[20] The Top 10s The James Lind Alliance: The James Lind Alliance. Available from: https://www.jla.nihr.ac.uk/top‐10‐priorities

[21] Jorge JM , Wexner SD . Etiology and management of fecal incontinence. Dis Colon Rectum. 1993;36(1):77–97. 8416784 10.1007/BF02050307

[22] Vaizey CJ , Carapeti E , Cahill JA , Kamm MA . Prospective comparison of faecal incontinence grading systems. Gut. 1999;44(1):77–80. 9862829 10.1136/gut.44.1.77 PMC1760067

[23] Taylor CJ , Huntley AL , Burden J , Gadoud A , Gronlund T , Jones NR , et al. Research priorities in advanced heart failure: James Lind Alliance priority setting partnership. Open Heart. 2020;7(1):e001258. 32606070 10.1136/openhrt-2020-001258 PMC7328807

[24] Finer S , Robb P , Cowan K , Daly A , Shah K , Farmer A . Setting the top 10 research priorities to improve the health of people with type 2 diabetes: a Diabetes UK–James Lind Alliance priority setting partnership. Diabet Med. 2018;35(7):862–870. 29485717 10.1111/dme.13613 PMC6032840

[25] Bang D , Frith CD . Making better decisions in groups. R Soc Open Sci. 2017;4(8):170193. 28878973 10.1098/rsos.170193 PMC5579088

