# Supplementary material for: Research priorities for faecal incontinence in adults: A James Lind Alliance priority setting partnership
Source: Colorectal Dis. 2025 Jul 10;27(7):e70154. doi: 10.1111/codi.70154 (PMC12246508; doi:10.1111/codi.70154)
Supplement: Supplementary file 1 — Data S1. [file CODI-27-0-s001.docx]

# Supplementary Tables

## Supplementary Table 1: Indicative summary questions included in the interim priority setting survey

| **Theme 1: Cause, diagnosis, disease course, linked conditions, prevention, triggers (15)** |
| --- |
| Cause1: How can the common causes of FI be better understood, predicted, and prevented? |
| Cause2: What is the role of lifestyle factors in causing faecal incontinence? *(e.g. obesity, mental health, and anal sex)* |
| Cause3: Are there any genetic factors in people having FI? |
| Cause4: How can FI from an injury during childbirth be predicted and avoided? |
| Cause5: What are the risks of FI in people with back problems or spinal injury? |
| D2: What are the most effective and acceptable tests to accurately understand the cause of FI, and inform relevant treatments? |
| DC1: How can worsening or deterioration of FI be predicted and prevented? *(e.g. due to aging or childbirth)* |
| DC4a: What part does the community of microbes in the gut (the gut microbiome) play in FI? |
| DC4b: What is the nature of the connection between the brain-gut axis and FI? *(the gut-brain axis is a two-way communication network that links the bowel and brain)* |
| DC5: Why does night-time leakage occur and what can be done to prevent it? *(e.g. routine, diet, sleep hygiene, and medications)* |
| DC9: What is the cause of flatus incontinence (uncontrolled passage of wind) and how can it best be managed? |
| Link 1: How is FI linked to menopause? |
| Prev3: In post-menopausal women, what is the role of topical HRT in preventing FI? *(e.g. creams, ointments, pessaries, and patches)* |
| Trig2: Which foods, supplements or medications are triggers for FI, and are these specific to each person? |
| Trig4: Why does FI seem to be triggered by stress and anxiety, and how can this be managed? |
| **Theme 2: Toilets (1)** |
| Toilets1: What is the impact of needing to access public toileting facilities frequently and/or urgently on people living with FI? |
| **Theme 3: Impact, experience and outcomes (12)** |
| E1: What would be the impact of recognising FI as a disability on the wellbeing of people living with FI? |
| E2: How can the individual experiences of people living with FI be better understood? |
| IM1: How can the psychological impact of FI be better understood? |
| IM2: What is the impact of FI on an individual’s social and day-to-day life? |
| IM4: What is the impact of FI on sex life and sexual partners? |
| IM5: What is the impact on family and those close to people living with FI, and how are they best supported? |
| IM6: What are the consequences for employment for people living with FI and how can they be best supported to work? |
| IM7: What is the financial burden to the people living with FI? *(e.g. cost of products, inability to use public transport, and laundry)* |
| IM8: What is the environmental impact of FI? *(e.g. disposable products and pad usage)* |
| IM9: What is the impact on people living with FI due to delays in being diagnosed, being misdiagnosed or being left untreated for their FI? |
| O1: What is the most effective method to measure FI symptoms and quality of life? *(e.g. to measure the response to treatment)* |
| O2: What are the economic and social benefits to the wider society of early recognition and treatment of FI? |
| **Theme 4: Awareness, Information, Education & Communications (12)** |
| A1: How can public awareness of FI be improved? |
| A3: What would be the impact if FI had the same public perception as urinary incontinence? |
| Com1: What are the implications and consequences of a lack of communication between different health care professionals supporting people living with FI and related issues? |
| Com2: What are the best ways to communicate and share up-to-date information with people living with FI? *(e.g. treatment options, products, and advice)* |
| Com3: How can the risks of FI associated with surgery, pelvic health, cancer treatment, and childbirth be effectively communicated to patients, so there is understanding and informed agreement? |
| Ed2: How may a healthcare professional’s limited awareness and understanding of FI impact the holistic relationship between the people living with FI and the healthcare professional? |
| Ed3: What are the shortfalls in the education of healthcare professionals with regard to management of FI, and how can this be improved? |
| Ed4: How are GPs and other primary health care professionals best trained and supported to provide best care for people living with FI? |
| Inf2: How can people living with FI be empowered to seek professional help and receive guidance to manage their condition in a safe and effective way? *(e.g. exercises, medications, dietary advice, and hydration)* |
| Inf3: What information should be given to women about risk of developing FI linked to pregnancy, childbirth, and postnatally, and how can this be effectively communicated? |
| SG1: What are the barriers to using the most up-to-date treatment guidelines and pathways for care of people living with FI? |
| SG2: How can provision of and access to FI services and products across the UK be standardised? |
| **Theme 5: Products (1)** |
| Pr1: How effective are current FI products (e.g. pads and plugs), and are they sufficiently adaptable for an individual’s needs? |
| **Theme 6: Support (4)** |
| S1: What are the personal difficulties or healthcare system limitations for people living with FI to seek help and support? *(e.g. cultural factors, embarrassment, or lack of information)* |
| S2: How can peer-support or support groups help people living with FI and their caregivers? |
| S3: What are the best ways of providing psychological support for people living with FI, and what are the barriers for accessing these? |
| S5: What are the barriers for people living with FI to timely access to an appropriate healthcare professional on a holistic treatment pathway? |
| **Theme 7: Treatments (9)** |
| T1: What are the most effective and acceptable treatment options for a given individual living with FI? |
| T2: What is the best approach for treatment and counselling where abuse has been involved for a person living with FI? *(e.g sexual, emotional, and physical abuse)* |
| T3: How effective are pelvic floor exercises to prevent, reduce or control FI? |
| T4: What is the role of neurostimulation/neuromodulation treatments in FI and faecal urgency? *(e.g. sacral nerve stimulation (SNS), or tibial nerve stimulation (PTNS/TTNS))* |
| T5: What new surgical techniques for FI could be developed and which existing surgeries could be improved to be less invasive? |
| T6: How can FI associated with severe injury during childbirth be better detected and treated? |
| T8: What new and novel approaches to managing FI could be developed? *(e.g. stem cells or bioelectronics)* |
| T9: What is the role of medications in treating FI, and how can the range of medications be improved? |
| T10: What are the most effective methods to prevent or control faecal urgency in people living with FI? |

## Supplementary Table 2: Combined rank of the 26 questions discussed at the final priority setting workshop

| **Rank** | **Question** |
| --- | --- |
| 1 | How are GPs and other primary health care professionals best trained and supported to provide best care for people living with FI? |
| 2 | How can the common causes of FI be better understood, predicted, and prevented? |
| 3 | How can the risks of FI associated with surgery, pelvic health, cancer treatment, and childbirth be effectively communicated to patients, so there is understanding and informed agreement? |
| 4 | How can people living with FI be empowered to seek professional help and receive guidance to manage their condition in a safe and effective way? (e.g. exercises, medications, dietary advice, and hydration) |
| 5 | What are the most effective and acceptable treatment options for a given individual living with FI? |
| 6 | How can worsening or deterioration of FI be predicted and prevented? (e.g. due to aging or childbirth) |
| 7 | What are the most effective methods to prevent or control faecal urgency in people living with FI? |
| 8 | What is the role of medications in treating FI, and how can the range of medications be improved? |
| 9 | How can the psychological impact of FI be better understood? |
| 10 | What part does the community of microbes in the gut (the gut microbiome) play in FI? |
| 11 | How effective are pelvic floor exercises to prevent, reduce or control FI? |
| 12 | How can provision of and access to FI services and products across the UK be standardised? |
| 13 | What are the most effective and acceptable tests to accurately understand the cause of FI, and inform relevant treatments? |
| 14 | What is the impact of FI on an individual’s social and day-to-day life? |
| 15 | What new surgical techniques for FI could be developed and which existing surgeries could be improved to be less invasive? |
| 16 | What is the nature of the connection between the brain-gut axis and FI? (the brain-gut axis is a two-way communication network that links the bowel and brain) |
| 17 | In post-menopausal women, what is the role of topical HRT in preventing FI? (e.g. creams, ointments, pessaries, and patches) |
| 18 | What would be the impact of recognising FI as a disability on the wellbeing of people living with FI? |
| 19 | What is the role of neurostimulation/neuromodulation treatments in FI and faecal urgency? (e.g. sacral nerve stimulation (SNS), or tibial nerve stimulation (PTNS/TTNS)) |
| 20 | What new and novel approaches to managing FI could be developed? (e.g. stem cells or bioelectronics) |
| 21 | Which foods, supplements or medications are triggers for FI, and are these specific to each person? |
| 22 | What is the role of lifestyle factors in causing faecal incontinence? (e.g. obesity, mental health, and anal sex) |
| 23 | Why does FI seem to be triggered by stress and anxiety, and how can this be managed? |
| 24 | What is the impact of needing to access public toileting facilities frequently and/or urgently on people living with FI? |
| 25 | How effective are current FI products (e.g. pads and plugs), and are they sufficiently adaptable for an individual’s needs? |
| 26 | What is the cause of flatus incontinence (uncontrolled passage of wind) and how can it best be managed? |
